# A potential outcomes approach to defining and estimating gestational age-specific exposure effects during pregnancy

**DOI:** 10.1177/09622802211065158

**Published:** 2022-01-05

**Authors:** Mireille E Schnitzer, Steve Ferreira Guerra, Cristina Longo, Lucie Blais, Robert W Platt

**Affiliations:** 1 Faculty of Pharmacy, 5622 Université de Montréal, Canada; 2 Department of Social and Preventive Medicine, 5622 Université de Montréal, Canada; 3 Department of Epidemiology, Biostatistics and Occupational Health, 5620 McGill University, Canada; 4 1234 Academisch Medisch Centrum Universiteit van Amsterdam, the Netherlands; 5 Hôpital du Sacré Coeur de Montréal, Centre intégré universitaire de santé et de services sociaux du Nord-de-l’île-de-Montréal, Canada; 6 Research Institute of the McGill University Health Centre, Canada

**Keywords:** Causal inference, counterfactuals, perinatal epidemiology, pregnancy exposures, targeted maximum likelihood estimation, target trials, inverse probability weighting, G-computation

## Abstract

Many studies seek to evaluate the effects of potentially harmful pregnancy exposures during specific gestational periods. We consider an observational pregnancy cohort where pregnant individuals can initiate medication usage or become exposed to a drug at various times during their pregnancy. An important statistical challenge involves how to define and estimate exposure effects when pregnancy loss or delivery can occur over time. Without proper consideration, the results of standard analysis may be vulnerable to selection bias, immortal time-bias, and time-dependent confounding. In this study, we apply the “target trials” framework of Hernán and Robins in order to define effects based on the counterfactual approach often used in causal inference. This effect is defined relative to a hypothetical randomized trial of timed pregnancy exposures where delivery may precede and thus potentially interrupt exposure initiation. We describe specific implementations of inverse probability weighting, G-computation, and Targeted Maximum Likelihood Estimation to estimate the effects of interest. We demonstrate the performance of all estimators using simulated data and show that a standard implementation of inverse probability weighting is biased. We then apply our proposed methods to a pharmacoepidemiology study to evaluate the potentially time-dependent effect of exposure to inhaled corticosteroids on birthweight in pregnant people with mild asthma.

## 1. Introduction

Due to ethical concerns regarding the randomization of drug usage in pregnant individuals (including cis women and trans people), the safety of drug exposures for perinatal outcomes is typically evaluated using observational data.^1,2^ Researchers are often interested in establishing the potential of a drug to impede growth or produce physical or functional defects in the embryo or fetus. Notably, effects may vary by the duration of the exposure in addition to the specific gestational periods in which the exposure took place.^2^ Thus, interest lies in evaluating exposure effects over the duration of the pregnancy and in identifying periods where drugs may be potentially harmful.

Over the past 35 years, major developments have improved the analysis of longitudinal exposures.^3–5^ In particular, in situations where time-varying confounders of a time-varying exposure may themselves be affected by previous exposure states, standard regression methods fail to estimate a parameter that is interpretable as a causative mechanism. In 1997, Robins proposed marginal structural models,^6^ which are models for outcomes under an imposed (counterfactual) exposure history, as an intuitive way to address this problem. Inverse probability weighting (IPW),^4^ which involves modeling the probabilities of exposure and censoring at a series of time points,^7^ was presented as an accessible method for estimating the parameters of such models. G-computation^3,8^ is another estimation approach relying on time-dependent modeling of the outcome. More robust approaches that require modeling of the exposures, censoring states, and outcomes were subsequently developed.^6,9,10^ In particular, targeted maximum likelihood estimation (TMLE),^11–13^ which builds off of previous theory and methods,^9,10^ is a framework for the construction of plug-in estimators that are typically doubly robust and locally efficient.

Recently, Hernán and Robins^14^ detailed the “target trials” approach, which is a cogent approach to the causal inference of longitudinal exposures that directly relates observational cohort design and exposure definitions to a hypothetical randomized controlled trial (RCT).^5^ The result is that the parameters estimated in the analysis of observational data can be clearly linked to those in an RCT with possibly time-varying treatment assignments and adherence. In particular, they propose to deal with patients in the study becoming contraindicated to treatment over time by defining “treatment strategies” that assign treatment unless the contraindication is present. This allows for the definition of pragmatic treatment effects that are relatable to real clinical practice.

In this study, we propose new parameters that represent longitudinal effects of drug exposure during pregnancy using the target trials approach. Our parameters are well-defined when the outcome can be defined at all possible delivery times given the cohort structure. In particular, we define the treatment strategy “assign the drug during a specific gestational period unless delivery has already occurred.” The strategy “discontinue the drug during a specific gestational period unless delivery has already occurred” is similar. Such strategies are well-defined in the sense that they could be employed in a hypothetical RCT. We define and interpret intent-to-treat and the so-called “sustained treatment” parameters related to this approach. We propose specific implementations of IPW, G-computation, and TMLE for the estimation of such parameters and verify their performance for the intent-to-treat parameter in a simulation study. Finally, we apply these methods in an application addressing the impact of low-dose inhaled corticosteroid (ICS) during pregnancy on birthweight for pregnant individuals with mild asthma.

## 2. Challenges in the analysis of pregnancy exposures

Our example relates to the usage of ICSs by asthmatic individuals during pregnancy. We are interested in evaluating the gestational age-specific effect of such exposure on birthweight. For mild asthma, treatment typically alternates between low daily doses of ICSs or no controller medication^15^. During pregnancy, individuals often reduce or discontinue usage of ICS though it is not medically recommended to do so.^16^ While uncontrolled asthma is known to be dangerous during pregnancy, previous investigations did not identify any safety concerns related to the use of low-dose ICS on pregnancy outcomes.^17^ However, few longitudinal analyses adjusting for time-dependent confounding have been conducted.^18^ To address this gap, a cohort of pregnant asthmatic individuals with deliveries between 1998 and 2008 was established from a linkage of administrative healthcare data from the province of Québec, Canada. ^19^

**Figure 1.**
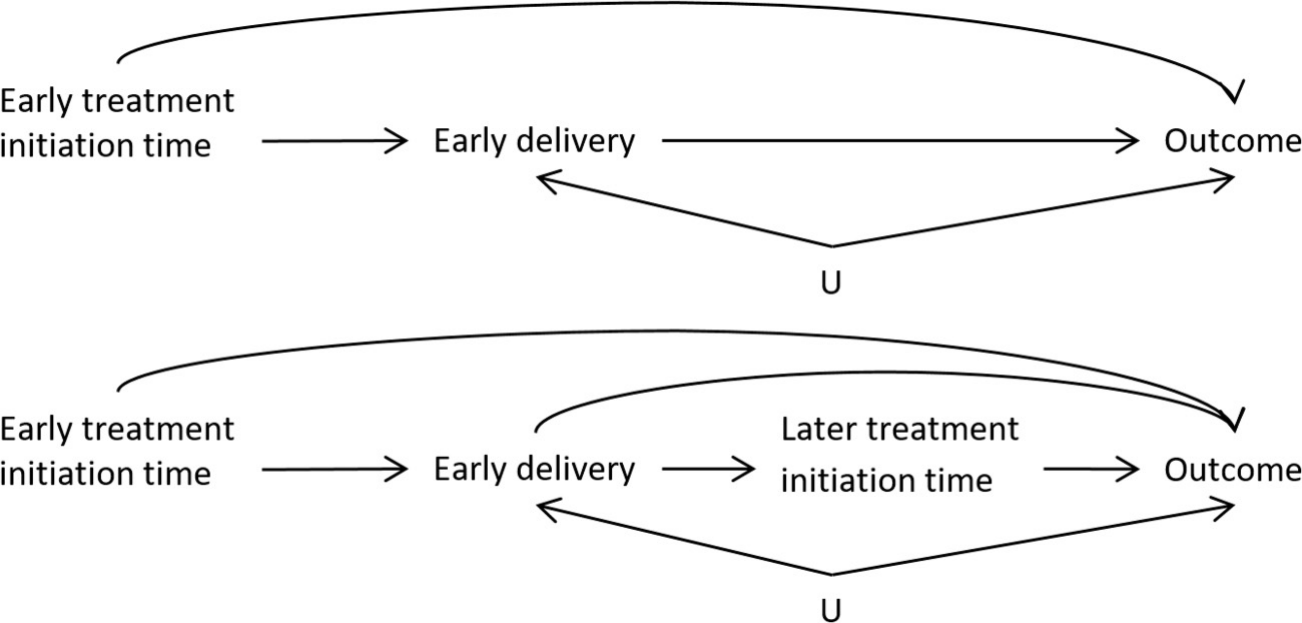
The approximate mediation structure of the hypothetical RCT where treatment initiation is randomized. (Top) The total effect of early treatment initiation may be divided into the mediated effect on early delivery and the direct effect on the outcome. U indicates unmeasured common causes of early delivery and the outcome. (Bottom) If we are also interested in later treatment effects, early delivery is both a mediator and a variable that prohibits future treatment initiation.

A general challenge in the analysis of time-varying drug exposures during pregnancy is that the duration of pregnancy is not the same for every person, and in fact may be related to past exposure and/or the outcome of interest when the outcome of interest is not the duration of the pregnancy itself.^20^ Due to the variability in delivery times, delivery may precede (compete with) exposure at later time points, producing a violation of the standard positivity assumption that all subjects must be eligible to follow all exposure sequences of interest.^7^ Thus, delivery time is both a mediator of early exposure and a contraindication/confounder for later exposure. This structure is represented in a simplified directed acyclic graph in Figure 1. The top diagram shows how the timing of delivery (e.g. early delivery: true/false) can be a mediator of early exposure to treatment and the outcome at delivery. The bottom diagram shows how, if a delivery does not occur prematurely (early delivery=false), treatment exposure can also occur in the late stages of pregnancy and potentially impact the outcome.

We are interested in estimating the total effect of timing-specific exposure to low-dose ICS on birthweight, which is the combination of the effect mediated by pregnancy duration and the direct effect which is not mediated by pregnancy duration. One potential way to address the problem of variable pregnancy duration is to artificially censor all pregnancies that do not reach full term. However, this approach would only estimate the direct effect on the outcome and not the total effect. Another typical approach used in pharmacoepidemiology contrasts pregnancies that were “ever exposed” with those “never exposed.” In addition to not addressing the time-dependent nature of the effects of exposure, this approach is subject to immortal time bias,^21^ exposure misclassification, and unadjusted time-dependent confounder bias.^2^

## The target trial

The general target trials approach^14^ involves the elaboration of an ideal RCT (target trial) that relates to the same scientific question as the observational study to be conducted. One must specify, for example, eligibility criteria, the baseline (when randomization occurs), treatment assignment groups, the follow-up period, and outcomes. To avoid selection bias in the RCT analysis, all subjects in the study would be retained, regardless of health or pregnancy status after baseline. The RCT parameters of interest, such as the intent-to-treat contrast, are then defined. Once the target trial is defined, the cohort design and analysis must be undertaken using the same definitions as the RCT, or as close as possible. The analysis of the observational data is then defined to estimate the same parameters as in the target trial.

For our example, we consider the hypothetical RCT that enrolls participants at conception. Each participant is then assigned a randomly selected start-time of ICS treatment. For example, we may enroll individuals with mild asthma at conception, all indicated for low-dose ICS, stop their current ICS usage, and assign them to a time to (re)start ICS. We consider four treatment arms with different start-times—conception, 13 weeks gestational age (beginning of second trimester), 28 weeks (beginning of third trimester), and 37 weeks—and a fifth control group that is assigned to never initiate. We also simplify reality so that no pregnancies terminate before 13 weeks and all will have a measurable outcome at delivery, such as weight at birth. In our RCT, it is possible that a delivery will occur before 37 weeks (11% globally) or even before 28 weeks (roughly 6 ‰ ).^22^ Thus, a woman randomized to the intervention “initiate treatment at week 37” may deliver prior to week 37 and thus never have the opportunity to initiate the treatment as assigned.

What are the options for the analysis of such an RCT if the outcome is binary, such as low birth weight, or continuous, such as birthweight? Analyses of RCTs often include the per-protocol and the intent-to-treat (ITT) approaches. In this section we discuss the potential for bias under a naive per-protocol analysis, the interpretation of the ITT effect in the described target trial, and the sustained treatment strategy parameter as an alternative to the naive per-protocol approach.

### 2.1 The fallacy of the naive per-protocol analysis

The naive per-protocol analysis involves the removal of all subjects who did not follow the assigned treatment protocol and estimates the treatment effect by making unadjusted comparisons between those remaining in each treatment arm. As is well-known, this type of analysis may be highly biased with respect to treatment efficacy if the reasons for deviation from the protocol are related to the outcome.^23^ Some authors have alternatively proposed to use measured time-dependent covariates to adjust for non-adherence post-randomization, in order to estimate the average effect of following the protocol.^24–27^ This approach requires that all relevant covariates are measured in order to adjust for confounding of the time-dependent protocol adherence. However, even if we did measure these covariates, we would not be able to apply this method in our RCT as it relies on the “positivity” assumption that all participants could have hypothetically followed any treatment protocol, conditional on their health status at each time point.^7^ In our case, pregnancies cannot become exposed to treatment after delivery, so we violate the assumption that any unit (pregnancy) could have followed any protocol.

In our context, a simple per-protocol analysis may also cause bias even in the absence of confounding. Consider an RCT that randomizes pregnant people 1:1 at conception either to initiating the medication at 37 weeks or control (never initiate). For illustration, suppose also that 10% of deliveries occur before 37 weeks. The outcome in this analysis is low birth weight (LBW) (<2500 g). Suppose that the medication has no biological effect on the outcome but that the 10% of early deliveries have a probability of LBW of 50% while full-term births have a probability of 5%. The initiation of treatment was randomized so half of the individuals with full-term births took the medication as assigned. However, due to early delivery, none of the early-term deliveries were exposed, despite 50% of them having been randomized to treatment. The simple per-protocol analysis would show that, because all early deliveries are included in the unexposed group regardless of their assignment, more unexposed pregnancies resulted in LBW so that the study may incorrectly conclude that medication was beneficial (Table 1, top). Note that in this simple case where no treatment arm involves initiating treatment in the second trimester, we can perform the per-protocol analysis in the subset of full-term pregnancies and get the correct null result, but this will not be the case in general.

**Table 1.** Expected proportions ( p ) of early- and full-term deliveries and of LBW among exposed and unexposed pregnancies in the RCT contrasting no exposure to starting medication at 37 weeks.

*Per-protocol*	Exposed	Unexposed	Probability LBW
p Early delivery	0	0.18	0.5
p Full term	1	0.82	0.05
p LBW	0.05	0.132	
*ITT/treatment strategy*	Treated group	Control group	Probability LBW
p Early delivery	0.1	0.1	0.5
p Full term	0.9	0.9	0.05
p LBW	0.095	0.095	

LBW: low birth weight; RCT: randomized controlled trial; ITT: intent-to-treat.

**Table 2.** Summary of notation used for the observed and counterfactual data and the intent-to-treat (ITT) parameter of interest.

*Variable*	*Meaning*
k	When used as superscript, represents the treatment strategy: initiate treatment at time *k* unless already delivered.
For t=1,…,K	
W(t)	Measured covariates at time *t*.
A(t)	Treatment status at time *t*, binary.
D(t)	Delivery status at time *t*, binary.
$S^{k}(t)$	Indicator of following treatment initiation strategy *k* up to time *t*.
$T_{D}$	Observed time of delivery.
Y	Outcome, measured at delivery.
$T_{D}^{k}$	Counterfactual delivery time that we assume would have been observed had the person been assigned the treatment initiation strategy *k*.
$Y^{k}$	Counterfactual outcome that we assume would have been observed had the person been assigned the treatment initiation strategy *k*.
$E(Y^{k_{1}})-E(Y^{k_{2}})$	The ITT contrast on the difference scale comparing treatment initiation strategies $k_{1}$ versus $k_{2}$ .

### 2.2 The treatment strategy approach: ITT and sustained treatment effects

Assume that the outcome is definable in all subjects regardless of delivery time and survival post-delivery. In an ITT analysis, we would aggregate the outcomes of all participants assigned to each arm regardless of whether they followed the protocol of starting treatment at the assigned time. For example, a woman assigned to initiate treatment at 37 weeks but delivers at 28 weeks would still be included in the “start at 37 weeks” group. We could then estimate the effects of being assigned to initiate treatment at each time point by comparing the means or proportions of the outcome in participants assigned to treatment initiation at week 0 versus 13, at week 13 versus 28, at week 28 versus 37, and at week 37 versus control, respectively. In the lower section of Table 1 describing the simple example in the last section, we see that the ITT approach gives the correct conclusion that there is no effect of treatment started at 37 weeks.

The ITT analysis estimates the total effect of the “treatment strategy” of assigning a treatment start-time on the outcome. If the treatment may be initiated early on in pregnancy, this effect includes the component that is a result of potentially shifting the time of delivery, as shown in Figure 1. Thus, it is possible that even if there is no direct effect of the treatment on the outcome, if the treatment has an effect on early delivery and if early delivery has an impact on the outcome, there will be a non-null effect of treatment on the outcome. It is also possible that early delivery and certain outcomes share common causes U though these would not need to be known in order to estimate the total effect of treatment on the outcome. For example, early delivery and low birth weight may both be affected by major congenital malformations, but we would not need to adjust the ITT analysis to estimate the total effect of treatment. The variable U would need to be adjusted for if we were interested in estimating mediated effects such as the direct effect that does not pass through early delivery.

In the simple example of Table 1, we saw that performing the analysis with only full-term deliveries gave the correct (null) answer when we were only interested in the effect of late-term exposure. When we are interested in the effects of early-term exposure, performing the full-term subgroup analysis will not be a viable strategy. If early delivery is affected by previous treatment, the full-term analysis will ignore the component of the effect of early treatment that passes through the mediator early delivery (Figure 1, top). In addition, if there is an unadjusted common cause of early delivery and the outcome ( U ) then subsetting on full-term deliveries will create an artificial association between early exposure and the outcome, resulting in collider (or selection) bias (Figure 1, bottom). Collider bias is bias that is induced by conditioning on a variable (not having an early delivery) that is caused by two covariates (early treatment and U ) which creates the non-causal association between the two covariates.^28^ It is also possible that participants in the study will stop taking their assigned treatment at any time between assignment and delivery. Under non-adherence, the ITT effect represents the effect of assigning a treatment initiation time while allowing for a variable delivery time. However, we may also be interested in estimating the effect of sustained treatment^3^ allowing for treatment to be interrupted by delivery time. Under the sustained treatment strategy approach, a participant is considered to be adhering to their assignment if they follow the *strategy* of “initiate treatment at time k and continue to take treatment unless interrupted by delivery.” Thus, the corresponding parameter would represent contrasts between adhering to treatment strategies with different starting times. Estimating this parameter in an RCT would require classifying adherence as yes/no at every time point and measuring and adjusting for all confounders of post-initiation adherence and the outcome. There would be no need to adjust for variables affecting delivery time (i.e. supposed confounders of delivery time and the outcome). If this is possible, one can account for non-adherence while estimating the total effect of time-point-specific exposure.

In the next section, we define the ITT parameter under the treatment strategy approach in an observational study. The sustained treatment parameter is similar and defined in the Supplemental Materials. These parameters correspond to those we defined in the target trial.

## 3. Observational data and parameters of interest

In order to follow the target trials approach, the specifications of the ideal RCT must be used in the cohort design and analysis. For example, subjects in the observational study must have the same entrance criteria and prospective follow-up as in the RCT. If the observational data are collected for administrative purposes, as in our case, then this would involve a secondary selection of subjects “entering” the study when eligible and limiting the analysis to the resulting subset of person-time. If we consider weekly time-points t in the follow-up, numbered starting from the entrance into the study, each subject time-point can be classified as “exposed” or “unexposed” (denoted A(t) ) based on drug dispensation information in the administrative data. Covariates can be measured at baseline ( W(1) ) and potentially updated at each time-point ( W(t) for later times, t ). The analysis of the observational data would then use causal inference methods like IPW or TMLE to adjust for time-dependent confounding between the different treatment groups (those who have been observed to initiate treatment at time k unless already delivered) as defined in the RCT. Importantly, these methods only allow for the prospective usage of data, so adjustment for confounding at a given time-point can only use past data for each person.

Thus we consider an observational study where we have sampled independent and identically distributed data of the form ({W(t),A(t),D(t);t=1,…,K},Y) for each of n participants. W(t) represents the covariates measured for each subject at times t=1,…,K . The binary variables A(t),t=1,…,K , represent whether a participant was taking the treatment of interest at time t . At each time, W(t) precedes A(t) . The indicators of delivery D(t) indicate whether a participant has delivered by time t , so that D(t)=1 implies $D(t^{*})=1$ for $t^{*}>t$ . We define time K such that D(K−1)=0 for at least some subjects but D(K)=1 for all, that is, all subjects have delivered by the end of the study. Let $T_{D}$ correspondingly denote the observed time of delivery. If a participant delivers by time t , they are ineligible for further exposure, so if D(t)=1 we set $W(t^{*})=A(t^{*})=NA$ for $t^{*}>t$ where NA means not applicable. The outcome, Y , must be defined and measured for all pregnancies at delivery, regardless of delivery time. Outcomes where this is broadly possible include neonatal period survival, and Apgar score (with stillbirths given a score of 0).

Finally, we can define the variable $S^{k}(t)$ to indicate whether an individual is, up to time t , effectively following the treatment strategy k , corresponding to the intervention that assigns treatment at time k . For example, for a subject who has no exposure to treatment by time 2, $S^{2}(1)=S^{3}(1)=S^{3}(2)=S^{4}(1)=S^{4}(2)=1$ but $S^{1}(1)=S^{1}(2)=S^{2}(2)=0$ . As a second example, a subject who never initiated treatment but delivered at time t would have $S^{k}(t)=1$ for k>t since they were essentially following all strategies that initiated treatment after their delivery time. It is important to recognize that, at a given time, observed treatment patterns can be compatible with multiple treatment strategies.

### 3.1 ITT parameter and identifiability assumptions

**Figure 2.**
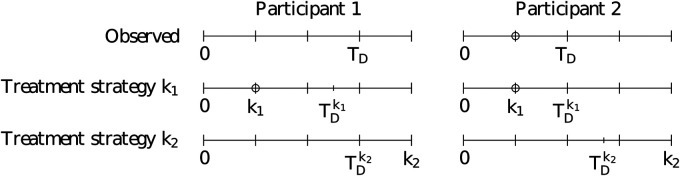
Observed and counterfactual timelines for two example study participants. The top row represents the observed delivery time while the second and third rows represent the counterfactual times under treatment strategies $k_{1}$ and $k_{2}$ , respectively. The circles represent the time where treatment is initiated. Note that participant 1 is adherent to strategy $k_{2}$ at all times ( $S^{k_{2}}(t)=1$ at all times t ) but only adherent to strategy $k_{1}$ prior to time $k_{1}$ since they were not observed to initiate treatment at $k_{1}$ ( $S^{k_{1}}(t)=1$ for $t<k_{1}$ ; 0 thereafter). Participant 2 is adherent to strategy $k_{1}$ at all times ( $S^{k_{1}}(t)=1$ at all times t ) but only adherent to strategy $k_{2}$ prior to time $k_{1}$ because they initiated treatment at that time ( $S^{k_{2}}(t)=1$ for $t<k_{1}$ ; 0 thereafter).

We are interested in defining the treatment effect corresponding to the ITT analysis (i.e. ITT parameter) in the hypothetical RCT ^5^. In order to define this parameter in the context of observational data, we first define the potential outcome $Y^{k}$ , corresponding to the outcome that an individual would have had if, at baseline, they had been assigned to treatment initiation at some fixed time k . We further define $Y^{K+1}$ as the potential outcome under the never-initiate-treatment strategy. While we do not augment the notation for simplicity, this potential outcome also depends on the potential delivery time, $T_{D}^{k}$ , that is, the delivery time that would have occurred if the individual had been assigned to treatment initiation at time k . Examples of observed and counterfactual delivery times are given in Figure 2. We can then define the parameter $E(Y^{k})$ as the expected outcome had we assigned the strategy with start-time k to all participants at baseline. We can define such a parameter for each time-point k=1,…,K+1 . Then, the ITT effect at time k versus k+1 can be defined as $E(Y^{k})-E(Y^{k+1})$ . If the outcome is binary, this effect would correspond to the ITT risk difference under the given counterfactual contrast.

Note that the potential outcomes defined above are different than the potential outcomes defined as “the outcome that an individual would have had had they started taking the treatment at time k .” In fact, under a given counterfactual scenario with initiation time k , if a participant would have delivered prior to k , they would not be eligible to start treatment at k . Therefore, potential outcomes defined in this way do not exist for all values of k and all subjects, making the corresponding parameters ill-defined.

In order to estimate the ITT effect from the observational data, we require additional assumptions. Firstly, we require “consistency,” meaning that if we observe that an individual initiated treatment at time k prior to delivering, their observed delivery time and outcome correspond to the counterfactual delivery time $T_{D}^{k}$ and outcome $Y^{k}$ , respectively, that would have been observed under the assignment of initiation time k . In addition, if a participant did not initiate treatment by their delivery time $T_{D}$ , then their observed delivery time and outcome are assumed to be equal to the counterfactual versions under any strategy that assigns treatment after time $T_{D}$ . These assumptions are present in Figure 2: when the counterfactual initiation time k is equal to the observed initiation time, then $T_{D}=T_{D}^{k}$ (participant 2); when k occurs after the observed delivery time for a participant who never initiated treatment, then $T_{D}=T_{D}^{k}$ (participant 1). Secondly, positivity in this case means that, conditional on the measured covariates (including delivery status) at a time point, all subjects who have not yet initiated treatment have a non-zero probability of following any treatment initiation strategy that involves starting in the future. This assumption could be violated if there are health status indicators that would contraindicate initiating treatment.^1^ In particular, by construction, once a delivery occurs at $T_{D}$ , a subject has a probability of one of continuing to follow all initiation strategies k for which $S^{k}(T_{D})=1$ . Thirdly, we require “exchangeability,” that all baseline and time-dependent confounders of the treatment initiation time and outcome have been measured,^7,3^ that is, for all times t , we require $Y^{k}⊥⊥A(t)|\bar{W}(t),\bar{A}(t-1)=0,\bar{D}(t-1)=0$ , where we use the notation $\bar{X}(t)=\{X(1),\ldots ,X(t)\}$ to denote the history of variable X up to time t . A violation of exchangeability occurs when unmeasured risk factors are also responsible for a participant’s treatment status, but this is only a concern prior to or at treatment initiation and prior to delivery. Finally, these potential outcomes are only well-defined in the absence of “interference,” meaning that one subject’s potential outcome cannot depend on the treatment of another.^29^ This assumption is violated in a vaccination effectiveness study where herd immunity protects unvaccinated participants from disease.

## 4. Estimation methods for observational data

In this section, we provide the algorithms to estimate the ITT parameter using IPW, G-computation, and TMLE. Corresponding estimators for the sustained treatment effect are given in the Supplemental Materials.

### 4.1 IPW

In a standard context where we would be interested in estimating the effect of a time-varying treatment, IPW involves the estimation of the conditional probability of treatment at each time point. The extension of IPW in this context involves the estimation of conditional probabilities of following the treatment strategy. The probability that must be estimated for each time t for a given treatment strategy starting at some time k (where k is a fixed value in 1,…,K ) or never ( k=K+1 ) is the probability of following the treatment strategy unless delivered, conditional on past adherence to the strategy. We can write these probabilities (for t=1,…,K ) as

$$\begin{array}{rl} & P(S^{k}(t)=1|\bar{W}(t),S^{k}(t-1)=1,\bar{D}(t-1))\\ = & \left\{ \begin{array}{cc} P(A(t)=I(k=t)|\bar{W}(t),\bar{A}(t-1)=0,D(t-1)=0) & \mathrm{if}\;t\leq k\;\mathrm{and}\;D(t-1)=0\\ 1 & \mathrm{otherwise} \end{array}\right. \end{array}$$

where the argument involving A(0) and $S^{k}(0)$ should be disregarded here and subsequently and defining D(0)=0 . These probabilities must be calculated for every subject.

To understand this probability at a given time t , we consider each of the time points t=1,…,K relative to the treatment strategy time k . For t=1,…,k−1 , if delivery has not yet occurred ( D(t−1)=0 ), we need to calculate the conditional probability of remaining untreated. If t=k and the delivery has not yet occurred, we need to estimate the conditional probability of initiating treatment at t . If t is past the start time k ( k<t ), or if delivery has already occurred ( D(t−1)=1 ), then the probability of continuing the strategy is automatically equal to one.

We thus see that we only need to estimate the probability of treatment for times prior to or equal to k , and at those times only for subjects who have not yet delivered. To do so, we may fit a regression model using the subset of subjects with D(t−1)=0 and who have not yet initiated treatment by time t−1 , conditional on the history of covariates $\bar{W}(t)$ . We can then estimate the probability for all subjects with D(t−1)=0 .

Once these probabilities are estimated, the IPW calculation for the effect of treatment initiation at k involves running an intercept-only linear regression for the outcome with weights $w_{n}^{k}(K)$ defined as estimates of $w^{k}(K)$ where

$$w^{k}(t)=S^{k}(t)\times [\prod_{l=1}^{t}P\{S^{k}(l)=1|\bar{W}(l),S^{k}(l-1)=1,\bar{D}(l-1)\}{]}^{-1}$$

The estimated intercept from the resulting model fit is the IPW estimate of the parameter $E(Y^{k})$ . This procedure upweights the outcomes of all subjects who followed the given strategy up to time K (i.e. $S^{k}(K)=1$ ), and assigns a weight of zero for subjects who did not follow the strategy throughout.

The populational means or risks $E(Y^{k})$ can be computed for different values of k and directly compared. Similarly one can fit a (generalized) linear regression model with binary covariates $S^{k}(K),k=2,\ldots ,K$ and weights $\sum_{k=1}^{K}w_{n}^{k}(K)$ . If the probability estimates converge at parametric (root- n ) rates, the confidence intervals for each contrast can be computed by bootstrap.^30^

### 4.2 G-computation

G-computation^3^ can also be used to estimate the effects of time-dependent treatments.^8,31^ Where IPW uses estimates of the probabilities of treatment at each time to estimate the target parameter, G-computation requires estimates of components related to the longitudinal outcome process. Here, we adapt G-computation to estimate the ITT parameter in our setting. The derivation and connection to a standard longitudinal procedure^31^ are given in the Supplemental Materials.

We need to estimate nested expectations of the outcome conditional on previously following the treatment strategy. We first define the expectation of the outcome, called ${\bar{Q}}_{K}^{k}$ , conditional on the history up until time K , including covariates and observed delivery status, and on following the treatment strategy up until time K . The nesting then involves defining an expectation of the previous expectation for each time t , conditioning on history and on following the treatment strategy up until time t .

Formally, we denote the nested expectations as ${\bar{Q}}_{t}^{k}$ for t=1,…,K+1 and a fixed k . First, initialize ${\bar{Q}}_{K+1}^{}=Y$ . Then, ${\bar{Q}}_{t}^{k}$ for t=K,…,1 can be written as

(1) $$\begin{array}{rl} & \left\{ \begin{array}{cc} D(t-1)Y+\{1-D(t-1)\}E\{{\bar{Q}}_{t+1}^{k}|D(t-1)=0,\bar{W}(t),\bar{A}(k-1)=0,A(k)=1\}\; & \mathrm{if}\;t\geq k\\ D(t-1)Y+\{1-D(t-1)\}E\{{\bar{Q}}_{t+1}^{k}|D(t-1)=0,\bar{W}(t),\bar{A}(t)=0\}\; & \mathrm{otherwise} \end{array}\right. \end{array}$$

We note that, by construction, no deliveries have occurred at the first time point so that D(0)=0 , and all deliveries have occurred by the final time point so that D(K)=1 .

The above equation shows that if delivery has occurred by t−1 (i.e. D(t−1)=1 ), the outcome Y is known at that time, so the (nested) expectation of the outcome is equal to the outcome. If delivery has not yet occurred (i.e. D(t−1)=0 ), then we need an estimate of the expectation. The conditioning statement in the expectations includes having followed the treatment strategy up until time t . If t<k , this means having not yet initiated treatment. If t≥k , then it means only initiating at time k .

We may model the expectations in equation (1) by regressing the predictions ${\bar{Q}}_{t+1}^{k}$ on covariate and treatment history amongst those who have not yet delivered at time t−1 . We obtain the estimates of ${\bar{Q}}_{t}^{k}$ by then taking the predictions from the regression model fit and setting $\bar{A}(k-1)=0$ and, if k<t , also setting A(k)=1 . The predictions are made for all subjects who have not yet delivered. For those who have delivered by t−1 , their estimate of ${\bar{Q}}_{t}^{k}$ is Y .

The estimation is iterative: take the vector of the predictions of ${\bar{Q}}_{t+1}^{k}$ and regress these values on the covariate history up until time t . The prediction from this new model fit are the estimates of ${\bar{Q}}_{t}^{k}$ . At t=1 , take the mean over the estimates of ${\bar{Q}}_{1}^{k}$ to obtain the G-computation estimate of $E(Y^{k})$ .

### 4.3 TMLE

TMLE^11,12^ uses the information from the probability of treatment weights to adjust the components ${\bar{Q}}_{t}^{k}$ estimated in the G-computation. Van der Laan and Gruber^13^ developed a TMLE framework for longitudinal treatments and we apply their general approach. For the estimation of $E(Y^{k})$ , our modification involves only making this adjustment to the random components of ${\bar{Q}}_{t}^{k}$ (i.e. the parts with the expectation in equation (1)), which is for subjects who have not yet delivered, and then reintegrating the known outcomes. We demonstrate the algorithm using an example below. The general algorithm is given in the Supplemental Materials and it is accessible to a reader with knowledge of the usual longitudinal TMLE algorithm.

Suppose that we only consider two time points so that our data are of the form (W(1),A(1),D(1),W(2),A(2),Y) with binary Y . We are interested in estimating $E(Y^{k=2})$ . The first step is to estimate the weights $w^{k}(1)$ and $w^{k}(2)$ , noting that k=2 . The probability of following the treatment strategy at the first time point is P(A(1)=0∣W(1)) ; for the second time point, it is P(A(2)=1∣W(1),W(2),A(1)=0,D(1)=0) if the delivery has not yet occurred ( D(1)=0 ) and equal to one if D(1)=1 . The probabilities can be estimated using a logistic regression, for example, or a more flexible modeling strategy. For the first time point weights, we have that $w^{k}(1)=(1-A(0))\times P(A(1)=0|W(1){)}^{-1}$ . For the second time point, $w^{k}(2)$ is equal to $w^{k}(1)\times A(1)\times P(A(2)=1|W(1),W(2),A(1)=0,D(1)=0{)}^{-1}$ for those with D(1)=0 , and equal to $w^{k}(1)$ for those who delivered ( D(1)=1 ).

For the first component of the G-computation used in the TMLE, we estimate the expectation part of equation (1) by regressing Y on W(1) , W(2) , A(1) , and A(2) in the subset of subjects who had not yet delivered at t=1 ( D(1)=0 ). This can incorporate a logistic regression model or a more flexible method. Setting A(1)=0 , and A(2)=1 , which corresponds to the strategy k=2 , we make a prediction for all subjects with D(1)=0 . Now we use the TMLE update step to incorporate the weights. Using the logit of the above predictions as an offset, we run an intercept-only logistic regression of Y with weights $w_{n}^{k}(2)$ in the subset of subjects with D(1)=0 . We then use this model to generate “updated” predictions, denoted ${\bar{Q}}_{2,n}^{k}$ for all subjects with D(1)=0 . The remaining subjects with D(1)=1 are given a prediction equal to their value of Y . The second component of the G-computation can then be estimated; this involves regressing ${\bar{Q}}_{2,n}^{k}$ on W(1) and A(1) . Setting A(1)=0 , this regression is then used to make predictions for all subjects. A second TMLE update step uses the logit of these predictions as an offset in an intercept-only logistic regression of ${\bar{Q}}_{2,n}^{k}$ with weights $w^{k}(1)$ . Predictions from this logistic regression are denoted ${\bar{Q}}_{1,n}^{k}$ . The mean of ${\bar{Q}}_{1,n}^{k}$ taken over all subjects is the TMLE estimate of $E(Y^{k=2})$ .

The benefits of using TMLE include double robustness, local efficiency, and the possible integration of machine-learning for the components that need to be estimated. Double robustness here means that if all of the estimators for the probabilities of treatment *or* if all of the estimators of the ${\bar{Q}}_{t}^{k}$ s are consistent in the sense that the corresponding IPW or G-computation estimator (respectively) is consistent, then the TMLE will be consistent. Local efficiency means that if all model components are correctly estimated, TMLE attains the minimal variance bound among regular asymptotically linear estimators making the same assumptions on the likelihood (often essentially limited to the time-ordering of variables). Parametric estimators may have lower variance but they make more modeling assumptions, possibly leading to estimation bias. Finally, because the convergence of TMLE depends on second-order error terms (unlike G-computation or IPW) it can incorporate estimators of the treatment and outcome models that converge at slower than parametric rates. While regularity conditions apply, some machine learning methods can be used to estimate these components.^32^.

## 5. Simulation study

**Figure 3.**
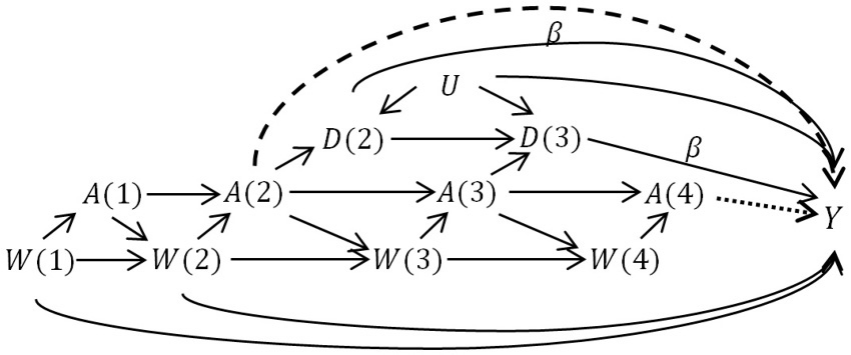
The data-generating structure in the simulation study. In scenario 1, odds ratio β=1 . In scenarios 2–4, odds ratio β=2 . The dashed arrow (effect of A(2) on Y ) occurs only in scenario 3. The dotted arrow (effect of A(4) on Y ) occurs only in scenario 4. We omitted the arrows indicating that when D(2)=1 , W(3)=A(3)=W(4)=A(4)=NA and when D(3)=1 , W(4)=A(4)=NA .

We conducted a simulation study in order to evaluate these three estimators implemented under the treatment strategy approach for estimating longitudinal effects during pregnancy and to compare with a standard implementation of IPW. We simulated independent and identically distributed observational data of the form (W(1),A(1),W(2),A(2),D(2),W(3),A(3),D(3),W(4),A(4),Y) , with respect to the directed acyclic graph in Figure 3, where each variable is binary. We first generated a completely random covariate W(1) and unmeasured common cause of early delivery and outcome, U . We allowed the probability of A(1)=1 to depend on the value of W(1) . For t>1 , the probability of W(t)=1 depended on the values of A(t−1) and W(t−1) . For simplicity, we generated perfect adherence to sustained treatment so that if A(t−1)=1 , the probability that A(t)=1 was one. If A(t−1)=0 , the probability that A(t)=1 depended on W(t) . For t=2 and 3, the probability of delivering D(t) depended on A(t) , and on U . If D(2)=1 then D(3)=1 . As before, if D(2)=1 then A(3)=A(4)=W(3)=W(4)=NA and if D(3)=1 then A(4)=W(4)=NA . Finally, the probability of the outcome Y=1 depended directly on W(1) , W(2) , and U .

Our goal was to estimate effects of exposure in the time intervals 2-3, 3-4, and 4+, corresponding to the ITT parameters $E(Y^{k=2})-E(Y^{k=3})$ , $E(Y^{k=3})-E(Y^{k=4})$ , and $E(Y^{k=4})-E(Y^{k=5})$ , respectively. Recall that $E(Y^{5})$ is the expected outcome under no exposure. Thus, in the first scenario, since the outcome was not affected by delivery time or exposure, each total effect was null. An example of such an outcome would be trisomy 21, which is the result of a genetic abnormality. In a second scenario, we also allowed the probability of the outcome to depend on the delivery time, where earlier delivery led to greater risk, with effect β=2 on the odds ratio scale (see Figure 3). An example of such an outcome is low birth weight. Even without direct effects of the exposure, this structure implies non-null effects of treatment due to the indirect effect passing through delivery time. In the third and fourth scenarios, we again set β=2 , and also let the risk of the outcome increase when either A(2)=1 (scenario 3) or A(4)=1 (scenario 4). We will interpret this as meaning that, in addition to the indirect effect through delivery time, we allowed for a direct effect of treatment in early or late-term pregnancy, in respective scenarios.

For each of 1000 repetitions, we drew n =1000 independent samples and used treatment strategy IPW, G-computation, and TMLE to estimate the three parameters of interest. We also used a “standard” implementation of IPW that weights outcomes of babies delivered full term (i.e. censors all outcomes with D(3)=1 ) by the inverse probabilities of observed exposure. This last implementation of IPW is problematic because, amongst individuals who are not exposed in the first two time periods, those who deliver early (with D(2)=1 or D(3)=1 ) will not be eligible for subsequent exposure. If these individuals have a different outcome distribution due to U or the delivery time, this approach will be biased.

The simulation results are presented in Table 3. In all four scenarios, the treatment strategy approaches were unbiased with similar standard errors. In the first scenario, there was no direct or indirect effect of treatment, so the true total effect of treatment at every time point was null. A standard implementation of IPW could be biased in this scenario because subsetting on the collider D(3)=0 induces a correlation between A(2) and U and between A(3) and U . In the simulation study, IPW was unbiased but had 65-90% greater standard errors than the treatment strategy approaches due to the inefficiency of subsestting on full term deliveries. In the second scenario, there were only indirect effects of treatment through delivery time, making the first two effects non-null. Standard IPW was biased, estimating null effects. This occurred because in subsetting on delivery time, standard IPW blocks the indirect effect of treatment. In the third scenario, there was a direct early effect of treatment in addition to the indirect effect of delivery time. The standard IPW produced bias, estimating a 50% attenuated effect in the 2-3 time interval and a null effect in the second time interval. The bias in the standard IPW is caused by subsetting on D(3)=0 which opens a backdoor biasing path through U in addition to blocking the indirect effects. In the fourth scenario, treatment had a true positive effect in all time intervals due to a direct effect of treatment in later pregnancy and indirect effects of delivery time. Standard IPW was again biased for similar reasons as in the third scenario.

**Table 3.** Bias and Monte Carlo standard errors for each estimator in the simulation study. Bias here is defined as the estimate - true value. 1000 repetitions of 1000 independent draws were taken. All estimators are for the ITT treatment strategy parameter except for “standard IPW.”

	$E(Y^{k=2})-E(Y^{k=3})$	$E(Y^{k=3})-E(Y^{k=4})$	$E(Y^{k=4})-E(Y^{k=5})$
*Scenario 1: No direct effect of treatment, no effect of delivery time*			
**True value**	0	0	0
IPW	− 0.001 (0.042)	− 0.001 (0.041)	0.000 (0.031)
G-computation	0.001 (0.040)	− 0.002 (0.040)	0.001 (0.029)
TMLE	− 0.001 (0.042)	0.001 (0.041)	0.000 (0.030)
Standard IPW	0.001 (0.077)	− 0.006 (0.069)	0.002 (0.050)
*Scenario 2: No direct effect of treatment, delivery time has effect*			
**True value**	0.028	0.027	0
IPW	0.003 (0.051)	− 0.003 (0.046)	− 0.001 (0.029)
G-computation	− 0.003 (0.051)	0.000 (0.043)	0.000 (0.029)
TMLE	0.001 (0.051)	− 0.001 (0.045)	0.000 (0.030)
Standard IPW	− 0.027 (0.079)	− 0.031 (0.069)	0.000 (0.051)
*Scenario 3: Direct effect of early treatment ( A(2) ), delivery time also has effect*			
**True value**	0.110	0.026	0
IPW	− 0.006 (0.054)	0.002 (0.045)	− 0.002 (0.030)
G-computation	− 0.002 (0.051)	− 0.002 (0.043)	0.000 (0.029)
TMLE	− 0.007 (0.053)	0.002 (0.046)	− 0.002 (0.030)
Standard IPW	− 0.061 (0.080)	− 0.029 (0.069)	− 0.001 (0.050)
*Scenario 4: Direct effect of late treatment ( A(4) ), delivery time also has effect*			
**True value**	0.025	0.012	0.032
TS-IPW	− 0.000 (0.051)	0.001 (0.048)	0.000 (0.035)
TS-G-computation	0.001 (0.050)	0.000 (0.047)	0.001 (0.031)
TS-TMLE	0.002 (0.051)	0.000 (0.048)	0.000 (0.034)
Standard IPW	− 0.027 (0.090)	− 0.016 (0.081)	0.021 (0.056)

## 6. Application

In this application, we aim to evaluate the time-period-specific effects of exposure to ICS versus no controller medication on birthweight in a population of pregnant individuals with mild asthma in the year prior to pregnancy. A limitation of this cohort is that, because it excludes all pregnancies that terminated prior to 20 weeks, this approach cannot directly estimate effects prior to 20 weeks gestation.^33^ We thus define our target trial as recruiting individuals at 20 weeks pregnancy who had not yet been exposed to ICS during the pregnancy and were diagnosed with mild asthma prior to pregnancy. In the hypothetical RCT, participants are then randomized to start ICS at 20, 26, 32, or 38 weeks and followed until the end of pregnancy, at which point the outcome, birthweight, is measured. The ITT effect is the effect of interest.

In our observational cohort, we subsetted to all individuals identified as having mild asthma who did not take ICS during the first trimester (prior to 12 weeks; this retained those who became exposed close to baseline at 20 weeks). We evaluated low-dose ICS usage at 20, 26, 32, and 38 weeks, denoted A(1),A(2),A(3) , and A(4) , respectively, defined as whether an active prescription of ICS overlaped the time point (yes/no). Baseline covariates were pregnant parent’s age at delivery, social assistance beneficiary status, rural residency, and asthma controlled in the year prior to pregnancy. Baseline and time-updated covariates, assessed in the time-intervals from conception-20 weeks, 20–26 weeks, 26–32 weeks, and 32–38 weeks, were chronic diseases of the parent: chronic hypertension, diabetes mellitus, other chronic diseases, and uterine disorders; pathologies related to pregnancy: gestational diabetes, gestational hypertension including pre-eclampsia and eclampsia, other pregnancy-related pathologies, and placental complications; and variables related to asthma control and severity: use of a short-acting beta-2 agonist, use of a leukotriene receptor antagonists, use of oral corticosteroids, use of intranasal corticosteroids, at least one hospitalization for asthma, and at least one emergency room visit for asthma. Censoring, resulting from diverging from one of the two treatment options (low-dose ICS or no ICS, both without long-acting beta-2 agonists), was assessed at 20, 26, 32, and 38 weeks, denoted C(1),C(2),C(3) , and C(4) , respectively. Delivery assessed in the intervals by 26, 32, and 38 weeks, and after 38 weeks, was denoted D(1),D(2),D(3) , and D(4) , respectively, and birthweight Y in grams was assessed at the time of delivery. The observed data structure was therefore ({W(t),C(t),A(t),D(t);t=1,2,3,4},Y) .

The cohort includes 2878 pregnancies. The baseline summary statistics of this cohort have been published elsewhere.^18^ Table 4 provides summary statistics for the number of censored pregnancies, distribution of deliveries, and exposure states over the four time points. Notably, only 10% of the cohort was censored by 38 weeks and roughly 80% of deliveries occurred after 38 weeks. There were also between 117 and 275 new exposures among those at-risk of delivering (i.e. uncensored and still pregnant), giving us a decent sample size to estimate each exposure model for IPW and TMLE. Finally, we can see that birthweight was strongly associated with delivery time.

**Table 4.** Censoring, deliveries, exposure to low-dose ICS, and mean birthweight over time in the pregnancy cohort of individuals with mild asthma.

Time point ( t ):	1	2	3	4
Censored—cum., *n*. (% of total)	177 (6.2)	222 (7.7)	269 (9.5)	288 (10)
Delivered—cum., *n*. (% of uncensored)	11 (0.4)	57 (2.1)	506 (19.4)	2590 (100)
Exposed—cum., *n*. (% of uncensored and at-risk of delivery)	275 (10.2)	465 (17.6)	624 (24.5)	619 (29.7)
Exposed—incident, *n*. (% of uncensored and at-risk of delivery)	275 (10.2)	200 (7.6)	177 (6.9)	117 (5.6)
Mean birthweight (g) of those delivered at time *t*	625.9	1205.1	2628.2	3239.4

**Figure 4.**
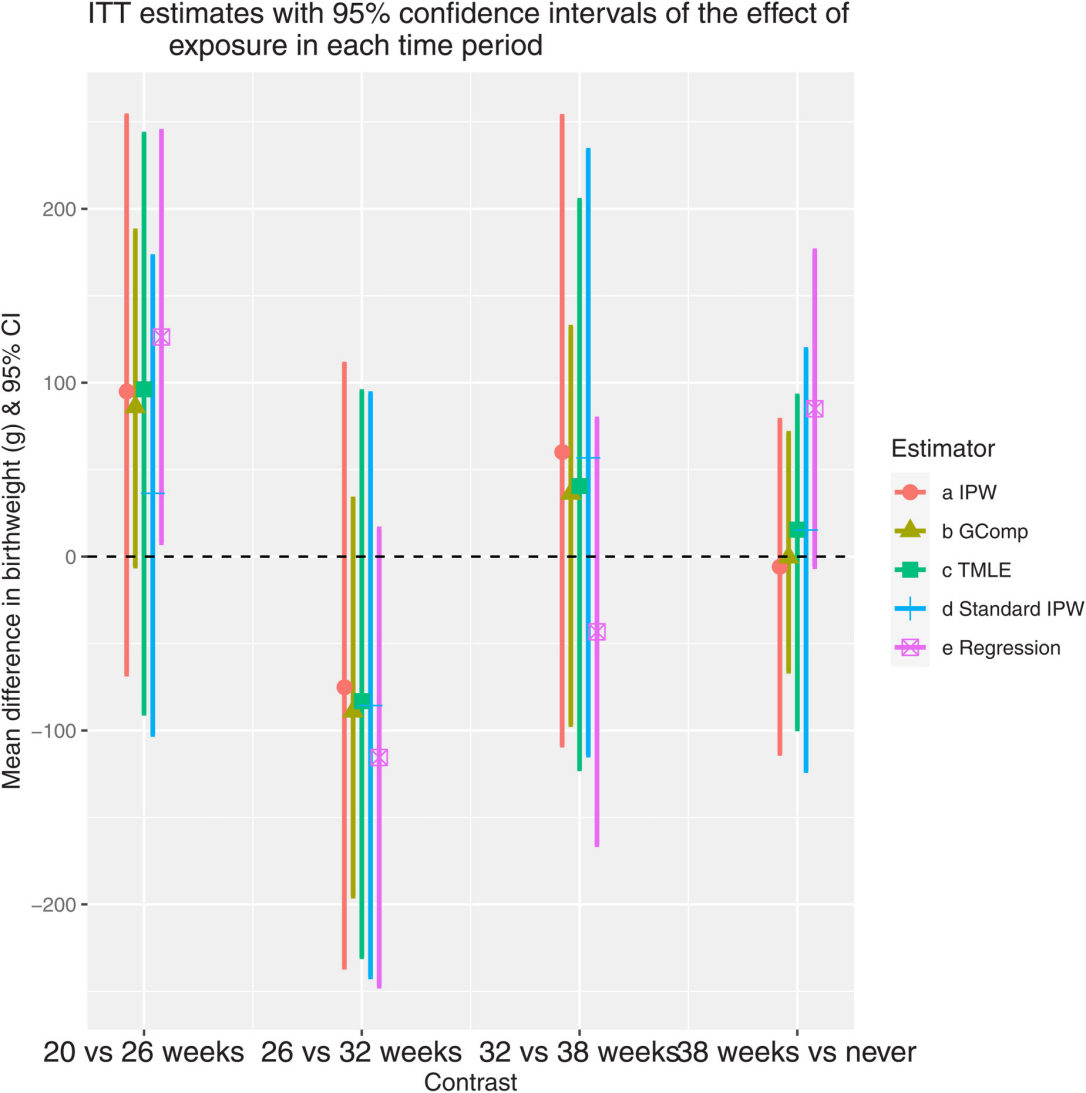
ITT estimates with 95% confidence intervals of the effect of exposure in each time period. The contrasts on the *x*-axis represent the comparison between different treatment initiation times. For the regression method, the estimates represent the corresponding coefficients of A(t),t=1,…,4 in a linear model, adjusting for confounders at all time-points. All confidence intervals were constructed by nonparametric bootstrap except for the regression results which use the profile likelihood.

We applied our IPW, G-computation, TMLE, and “standard” IPW as described previously to estimate the ITT parameters representing the effects of exposure at 20, 26, 32, and 38 weeks. We also used a naive linear regression model with only uncensored subjects, adjusting for exposure and confounders at all time points, and ignoring delivery time. For this model, all covariates were assigned a value of zero after the delivery time. Figure 4 presents the estimates and confidence intervals of the four time-specific effects for each estimator. The point estimates for all methods suggested a beneficial effect of ICS for the contrast $E(Y^{k=1})-E(Y^{k=2})$ ; this is interpreted as roughly a 100 g average increase in birthweight when ICS use starts at 20 versus 26 weeks. However, only the naive regression had a confidence interval that excluded the null. In contrast, all methods produced point estimates suggesting a harmful effect at 26 versus 32 weeks but all confidence intervals contained the null so we would not conclude that any safety concern exists given our data. The usage of the IPW estimator that censored early deliveries made a large difference for 20 versus 26 weeks but not otherwise. If there is indeed an effect of ICS in the earlier weeks, this suggests that a large portion of the effect may be mediated by delivery time.

## 7. Discussion

In this paper, we have proposed new parameters, based on the target trials approach, that represent total gestational age-specific effects of exposure during pregnancy when the outcome is binary or continuous. When the outcome of interest is gestational age at birth, a more standard survival analysis for time-dependent exposures is appropriate.^4^ The target trials approach avoids immortal time-bias and exposure misclassification by defining a common baseline and entrance criteria and by prospectively evaluating exposure status rather than grouping participants retrospectively according to a summary of their pregnancy exposures. We also avoid excluding eligible participants due to post-baseline health or pregnancy status by defining contrasts between treatment strategies that can be adhered to regardless of health or pregnancy outcomes during follow-up. This contributes to the definition of the contrast of interest and minimizes selection bias in the estimation.

The total effect that we defined includes the mediated portion of the effect that is the result of medication usage affecting delivery time. Because delivery time is strongly connected with birth outcomes,^34^ the total effect of medication exposure is of interest when evaluating medication safety. However, it may also be possible to perform mediation analysis to estimate the component of the effect that is mediated through delivery time and the direct effect of medication exposure on the outcome that is not related to changing delivery time. This would, however, require adjustment for all confounders of delivery time and a method that can incorporate time-varying exposures and mediator.^35^

We used a simple numerical example and directed acyclic graphs to demonstrate that naive per-protocol analysis and analyses that subset on full-term births are not appropriate for evaluating gestational age-specific effects of exposure. Other work has criticized methods that do not incorporate the time-varying nature of exposure.^21,2^ Our proposed parameters and methods allow for the estimation of the total effect of initiating treatment at different times (the ITT effect) and of initiating and sustaining treatment at different times (the sustained treatment effect).

We demonstrated the construction of an IPW estimator, a G-computation estimator, and a TMLE for the estimation of the ITT and sustained treatment effects. Each of these estimators requires the modeling of nuisance quantities, such as the probability of exposure at each time point and the nested conditional expectations of the outcome. Because of the added complication of variable delivery time, these quantities must be decomposed into random and constant components in order to propose reasonable modeling strategies. While the modeling or estimation of the random parts is flexible, standard semiparametric theory places restrictions on the regularity conditions and rates of convergence for the different types of estimators. In particular, among these, only TMLE can formally incorporate nonparametric modeling of these components, though some convergence rate restrictions apply.^36^

When designing an observational study, it is necessary to measure all confounders of the initiation time and outcome in order to fulfill the exchangeability assumption for the ITT parameter. If we are interested in estimating the sustained treatment parameter, we must also adjust for all confounders of post-initiation adherence. It is not necessary to measure all confounders of delivery time and outcome. This last fact was addressed in the simulation study where we included an unmeasured confounder of early delivery times and the outcome that had no impact on the unbiasedness of the proposed estimators. However, we also showed that a “standard” IPW implementation that censors early deliveries was substantially biased.

Finally, we demonstrated the application of our methods to evaluate the safety of inhaled corticosteroids during pregnancy, taking birthweight as the outcome of interest. While all confidence intervals for the proposed methods included the null, point estimates suggested a potential benefit of ICS at 20 weeks with evidence that much of the effect is mediated by the delivery time. We would typically expect that ICS use would lead to better control of asthma symptoms and reduce the risk of premature delivery, corresponding to a positive indirect effect through delivery time. Though we adjusted for asthma control indicators, there may have been residual confounding where individuals with more severe symptoms were more likely to use ICS, and this may have attenuated the estimate of the positive effect.

An important limitation of the applicability of these methods is that the outcome must be defined and measurable for all time points under study where delivery or pregnancy loss is possible. When the study baseline is prior to the second trimester this may be problematic as many outcomes, including birthweight, are not definable for early pregnancy losses. If we restrict the cohort to only include pregnancies that continue past a given threshold (e.g. baseline at 20 weeks as in the application) and thus restrict the delivery times under study to times where the outcome can be defined, we can evaluate exposure effects after the threshold. However, in such a restricted cohort, selection bias may hinder the estimation of exposure effects prior to the threshold.^37,33^ Some potential solutions to dealing with outcomes that cannot be defined for early pregnancy loss were proposed by Chiu et al. ^38^ in the context of an RCT evaluating fertility therapies. One possibility is to define composite outcomes that include the competing event of “no live birth,” though this doesn’t explicitly consider causal pathways. A second option incorporates separable direct and indirect effects where treatments are conceptually decomposed into the treatment component that impacts the occurrence of live birth and the component that impacts the primary outcome.^38^ A third option is to define exposure effects in the principal stratum of pregnancies that would always result in live birth, regardless of treatment.^39,40,38^ However, because it is difficult to identify pregnancy initiation and very early pregnancy losses, cohorts restricted to pregnancies that cross a time threshold are typical and selection bias omnipresent for the estimation of early exposure effects during pregnancy,^41^ though this will impact RCTs as well.

These parameters and methodological extensions are important contributions to the literature as they overcome the limitations of many standard methods used to evaluate the time-dependent impact of medication exposure during pregnancy using observational data. The parameters are interpretable and estimation is flexible, with multiple modeling options available. This work also allowed for insight into the specific assumptions that are required for consistent estimation of the parameters. Understanding these assumptions is important when designing studies and interpreting the results of the application of these methods. The uptake of such methods may thus facilitate more robust evaluations of medication safety during pregnancy using observational data.

## Supplemental Material

sj-pdf-1-smm-10.1177_09622802211065158 - Supplemental material for A potential outcomes approach to defining and estimating gestational age-specific exposure effects during pregnancy Supplemental material, sj-pdf-1-smm-10.1177_09622802211065158 for A potential outcomes approach to defining and estimating gestational age-specific exposure effects during pregnancy by Mireille E Schnitzer, Steve Ferreira Guerra, Cristina Longo, Lucie Blais and Robert W Platt in Statistical Methods in Medical Research

## Notes

1. Note that we could incorporate such contraindications into the treatment strategy as we did with early delivery. Hypothetically, we could avoid problems associated with positivity violations entirely as long as all contraindications are noted in the patient files.^43^
