# Supplementary material for: A potential outcomes approach to defining and estimating gestational age-specific exposure effects during pregnancy
Source: Stat Methods Med Res. 2022 Jan 5;31(2):300–14. doi: 10.1177/09622802211065158 (PMC8829732; doi:10.1177/09622802211065158)
Supplement: sj-pdf-1-smm-10.1177_09622802211065158 - Supplemental material for A potential outcomes approach to defining and estimating gestational age-specific exposure effects during pregnancy [file sj-pdf-1-smm-10.1177_09622802211065158.pdf]

Supplemental Materials for "A potential outcomes approach to defining and estimating gestational age-specific exposure effects during pregnancy"

Mireille E. Schnitzer[1,2,3,*], Steve Ferreira Guerra[3], Cristina Longo[4], Lucie Blais[1,5], Robert W. Platt[3,6]

[1]Faculty of Pharmacy, Université de Montréal, Montreal, Canada
[2]Department of Social and Preventive Medicine, Université de Montréal, Montreal, Canada
[3]Department of Epidemiology, Biostatistics and Occupational Health, McGill University, Montreal, Canada
[4]Academisch Medisch Centrum Universiteit van Amsterdam, Amsterdam, Netherlands
[5]Hôpital du Sacré Coeur de Montréal, Centre intégré universitaire de santé et de services sociaux du Nord-de-l'île-de-Montréal, Montreal, Canada
[6]Research Institute of the McGill University Health Centre, Montreal, Canada
[*]email correspondence: mireille.schnitzer@umontreal.ca

August 30, 2021

1 The sustained treatment effect parameter

In the main manuscript, we defined the intent-to-treat parameter to represent the effect of initiating treatment at a given point during a pregnancy where the pregnancy duration is allowed to be random and potentially precede the planned initiation. The sustained treatment effect is the effect of initiating and remaining on treatment from the given time point until the end of pregnancy.

1.1 Observed data

The observational data collected for each of n participants are independent and identically distributed (i.i.d.) and of the form $(W(t), A(t), D(t); t = 1, ..., K, Y)$. The full description is given in the main text, but briefly, $W(t)$ represents the covariates, $A(t)$ is the exposure, and $D(t)$ is the delivery status at time t. Time K is the first time point at which all subjects have delivered (i.e. $D(K) = 1$ for all but $D(K-1) = 0$ for some). Let T_D represent the observed time of delivery. Let an overbar refer to a history of a variable up to the indicated time point, e.g. $\overline{A}(k) = \{A(0), ..., A(k)\}$ if $k > 0$ and $\overline{A}(0) = A(0)$. Y is the outcome measured at delivery.

We define the indicator $\sigma^k(t)$ of sustaining strategy k up to and including time t as

$$\sigma^k(t) = \begin{cases} 1 & \text{if } T_D \leq k \text{ and } \overline{A}(k) = 0 \\ 1 & \text{if } k < T_D \text{ and } \overline{A}(k-1) = 0, A(k^*) = 1 \text{ for all } k^* \in \{k, min(t, T_D)\} \\ 0 & \text{otherwise} \end{cases}$$

which indicates whether a participant initiated treatment at time k and continued treatment until time t or were following this strategy until they delivered.

1.2 Sustained treatment strategy parameter and identifiability assumptions

Now we define the effect of initiating and sustaining treatment at time k until interrupted by delivery. The potential outcome under the sustained strategy Y^{σ^k} is the outcome that a participant would have had had they persisted in taking the assigned treatment from time k to K until interrupted by delivery. The parameter of interest is thus $E(Y^{\sigma^k})$ for each starting time k, allowing us to make contrasts between alternative start times.

In order to estimate the sustained treatment strategy effect with observational data, we require similar assumptions to that of the ITT parameter. Consistency here means that $Y = Y^{\sigma^k}$ if $\sigma^k(K) = 1$. As before, if a participant has not yet initiated treatment prior to a delivery time $T_D < k$, then their observed outcome is assumed to be equal to the counterfactual Y^{σ^k} for any $k \geq T_D$. Positivity here means that, conditional on the measured covariates (including delivery status) at a time point, all subjects would have a non-zero probability of continuing to follow any sustained treatment strategy at each time point. By construction, once a delivery occurs at T_D, the subject has a probability of one of continuing to follow all strategies k for which $\sigma^k(T_D) = 1$. Thirdly, we require a stronger type of exchangeability, that all baseline and time-dependent confounders of the exposure and outcome have been measured, i.e. $Y^{\sigma^k} \perp\!\!\!\perp A(t) \mid \overline{W}(t)), \overline{A}(t-1), \overline{D}(t-1) = 0$. This is a stronger assumption as we must measure the confounders of treatment taken at each time point rather than just the confounders of initiating treatment, amongst those who have not yet delivered. Non-interference is required as before.

2 More details about the estimation of the ITT parameter

2.1 G-computation

Firstly, by construction, no deliveries have occurred at the first time point ($D(0) = 0$) and all deliveries have occurred by the final time point ($D(K) = 1$). We then need to trivially modify our representation of the data

to include the outcome at every time point. If the measured outcome is Y, we define a time-varying Y to be unknown (NA) up until the delivery, after which it remains the same. Thus we initialize $Y(K+1) = Y$ and define $Y(t) = \{Y(t+1) \text{ if } D(t-1) = 1 \text{ or } NA \text{ otherwise}\}, t = K, ..., 2$, so that the complete data structure is $\{W(t), A(t), D(t), Y(t+1); t = 1, ..., K\}$.

Initializing $\overline{Q}_{K+1} = Y$, the estimator for treatment initiation can be defined through the nested expectations $\overline{Q}_t = E\{\overline{Q}_{t+1} \mid \overline{D}(t-1), \overline{Y}(t), \overline{W}(t), S^k(t) = 1\}, t = K, ..., 1$. We have that $E(Y^k) = E(\overline{Q}_1)$ under the causal assumptions. This corresponds to the standard decomposition of the exposition of the outcome expectation used in longitudinal TMLE; see Schnitzer et al [1] for more explanation.

The estimation is iterative: take the vector of the predictions of $\overline{Q}_{t+1}$ and regress these values on the covariate history up until time t. The prediction from this new model fit are the estimates of $\overline{Q}_t$. At $t = 1$, take the mean over the estimates of $\overline{Q}_1$ to obtain the G-computation estimate of $E(Y^k)$.

We decompose the $\overline{Q}_t$ expectations in order to develop reasonable modeling strategies. We note that

$$\overline{Q}_t = D(t-1)Y(t) + \{1 - D(t-1)\}E\{\overline{Q}_{t+1} \mid D(t-1) = 0, \overline{W}(t), S^k(t) = 1\}$$
$$= \begin{cases} D(t-1)Y + \{1-D(t-1)\}E\{\overline{Q}_{t+1} | D(t-1)=0, \overline{W}(t), \overline{A}(k-1)=\mathbf{0}, A(k)=1\} & \text{if } k \leq t \\ D(t-1)Y + \{1-D(t-1)\}E\{\overline{Q}_{t+1} | D(t-1)=0, \overline{W}(t), \overline{A}(t)=\mathbf{0}\} & \text{otherwise} \end{cases} \quad (1)$$

The above equation shows that if delivery has occurred by $t-1$, the outcome $Y = Y(t+1)$ is in the history and included in the conditioning statement, so the (nested) expectation of the outcome is equal to the outcome. If delivery has not yet occurred, then we need to model the expectation. For instance, we may model the expectations in the equation 1 by regressing the predictions $\overline{Q}_{t+1}$ on covariate and treatment history amongst those who have not yet delivered at time $t-1$. We obtain the estimates of $\overline{Q}_t$ by then taking the predictions from the regression model fit and setting $\overline{A}(k-1) = \mathbf{0}$ and, if $k < t$, also setting $A(k) = 1$, for all subjects who have not yet delivered. For those who have delivered by $t-1$, their estimate of $\overline{Q}_t$ is Y.

2.2 TMLE

Algorithm 1 Targeted Minimum Loss-Based Estimation for $E(Y^k)$

Initialize $\overline{Q}^*_{K+1,n} = Y$.

for $t = K, ..., 1$ **do**

Estimate $\overline{Q}_{t,1} = E\{\overline{Q}_{t+1} \mid \overline{W}(t), S^k(t) = 1, D(t-1) = 0\}$ for all subjects with $D(t) = 0$ by running a regression with outcome $\overline{Q}^*_{t+1,n}$ in the subset with $D(t-1) = 0$. Use this regression fit to make a prediction on the scale of the outcome for all patients with $D(t-1) = 0$. Denote this prediction by $\overline{Q}_{t,1,n}$.

"Update" these predictions by running a logistic regression of $\overline{Q}^*_{t+1,n}$ with an intercept term and offset $logit(\overline{Q}_{t,1,n})$ with weights $w^k_n(t)$ in the subset of patients with $D(t-1) = 0$. Denote the estimate of the intercept as $\hat{\epsilon}_t$.

Set $\overline{Q}^*_{t,1,n} = expit\{logit(\overline{Q}_{t,1,n}) + \hat{\epsilon}_t\}$, the updated estimate of $\overline{Q}_{t,1}$.

Set $\overline{Q}^*_{t,n} = D(t-1)Y + \{1 - D(t-1)\}\overline{Q}^*_{t,1,n}$, the updated estimate of $\overline{Q}_t$.

The final estimate is the mean of $\overline{Q}^*_{1,n}$ over all subjects.

3 Estimation of the sustained treatment effect parameter

3.1 IPW

The probabilities of following the treatment strategy to initiate and sustain treatment starting at time $k = 1, ..., K$ or never ($k = K + 1$) unless delivered are as follows:

$$P(\sigma^k(t) = 1 \mid \overline{W}(t), \sigma^k(t-1) = 1, \overline{D}(t-1))$$
$$= \begin{cases} P(A(t)=I(k \leq t) \mid \overline{W}(t), D(t-1)=0, \{A(l-1)=I(k \leq l-1); l=2,...,t\}) & \text{if } D(t-1) = 0 \\ 1 & \text{otherwise} \end{cases},$$

for $t = 1, ..., K$ where the argument involving $\sigma^k(0)$ is disregarded.

Once these probabilities are estimated, the IPW calculation for the effect of sustained treatment from time k involves running an intercept-only linear regression for the outcome with weights $w^k_{\sigma,n}(K)$ equal to estimates of $w^k_\sigma(K)$ where

$$w^k_\sigma(t) = \sigma^k(t) \times \left[\prod_{l=1}^{t} P\{\sigma^k(l) = 1 \mid \overline{W}(l), \sigma^k(l-1) = 1, \overline{D}(l-1)\} \right]^{-1}.$$

The estimated intercept from the resulting model fit is the IPW estimate of the parameter $E(Y^{\sigma^k})$.

3.2 G-computation

Recall that the complete data structure is $\{W(t), A(t), D(t), Y(t+1); t = 1, ..., K\}$.

For the sustained treatment effect, we define $\overline{Q}^{\sigma}_{K+1} = Y$ and $\overline{Q}^{\sigma}_t = E\{\overline{Q}^{\sigma}_{t+1} \mid \overline{D}(t), \overline{Y}(t), \overline{W}(t), \sigma^k(t) = 1\}, t = K, ..., 1$. The simplifications are the same as for the ITT setting, resulting in

$$\overline{Q}^{\sigma}_t = \begin{cases} D(t-1)Y + \{1-D(t-1)\}E\{\overline{Q}^{\sigma}_{t+1} | \overline{W}(t), \overline{A}(k-1)=\mathbf{0}, \underline{A}^t(k)=\mathbf{1}, D(t-1)=0\} & \text{if } k \leq t \\ D(t-1)Y + \{1-D(t-1)\}E\{\overline{Q}^{\sigma}_{t+1} | \overline{W}(t), \overline{A}(k-1)=\mathbf{0}, D(t-1)=0\} & \text{otherwise} \end{cases}$$

where we take $\underline{A}^t(k) = (A(k), ..., A(t))$ to indicate treatments from time k to t when $k \leq t$.

3.3 TMLE

The TMLE procedure for the sustained treatment effect follows essentially the same steps as the procedure for the ITT parameter.

Algorithm 2 Targeted Minimum Loss-Based Estimation for $E(Y^{\sigma^k})$

Initialize $\overline{Q}^{\sigma *}_{K+1,n} = Y$.

for $t = K, ..., 1$ **do**

Estimate $\overline{Q}^{\sigma}_{t,1} = E\{\overline{Q}^{\sigma}_{t+1} \mid \overline{W}(t), \sigma^k(t) = 1, D(t-1) = 0\}$ for all subjects with $D(t-1) = 0$ by running a regression with outcome $\overline{Q}^{\sigma *}_{t+1,n}$ in the subset with $D(t-1) = 0$. Use this regression fit to make a prediction on the scale of the outcome for all patients with $D(t-1) = 0$. Denote this prediction by $\overline{Q}^{\sigma}_{t,1,n}$.

"Update" these predictions by running a logistic regression of $\overline{Q}^{\sigma *}_{t+1,n}$ with an intercept term and offset $logit(\overline{Q}^{\sigma}_{t,1,n})$ with weights $w^k_{\sigma,n}(t)$ in the subset of patients with $D(t-1) = 0$. Denote the estimate of the intercept as $\hat{\epsilon}_t$.

Set $\overline{Q}^{\sigma *}_{t,1,n} = expit\{logit(\overline{Q}^{\sigma}_{t,1,n}) + \hat{\epsilon}_t\}$, the updated estimate of $\overline{Q}^{\sigma}_{t,1}$.

Set $\overline{Q}^{\sigma *}_{t,n} = D(t-1)Y + \{1 - D(t-1)\}\overline{Q}^{\sigma *}_{t,1,n}$, the updated estimate of $\overline{Q}^{\sigma}_t$.

The final estimate is the mean of $\overline{Q}^{\sigma *}_{1,n}$ over all subjects.

References

[1] Schnitzer ME, Van der Laan MJ, Moodie EEM et al. Effect of breastfeeding on gastrointestinal infection in infants: A targeted maximum likelihood approach for clustered longitudinal data. *Annals of Applied Statistics* 2014; 8(2): 703–725.
